# Mr. Fawell's Case of Superfœtation

**Published:** 1809-07

**Authors:** Thomas Fawell

**Affiliations:** Surgeon. Leeds


### *To the Editors of the Medical and Physical Journal.*

Gentlemen,

HAVING mentioned to some of my medical friends a Case of *Super-fœtation,* which occurred to me in my practice a few weeks ago, I was requested to state it to you for insertion in your periodical publication, which I leave to your judgment, if you should deem it worthy of notice.

On the 13th of April last, I was called to Mrs. Lacer, of Hunslett, near this place, in labour, who had previously spoken to me to attend her; on my entering into the room, I proceeded to examine her *per vaginam,* when I found the os uteri completely dilated, and the membranes unbroken; the labour went on regularly, and in the course of half an hour, one living child was expelled.—After having separated the child, I laid my hand (as usual) upon the abdomen, men, and found the uterus not contracted; after a few minutes, a pain came on, when I examined her again, and found the membranes pressing forwards, and in the course of half an hour, she was delivered of a fœtus; I should suppose from its size, about 4 or 5 months. Apparently it had been dead a few days; it had no putrid appearance, but rather inclining to a darkish hue. The woman recovered remarkably well, and is in perfect health.

I should be happy if any of your readers would favour me with a few observations upon the above case.

I am, &c.

THOMAS FAWELL, Surgeon.

*Leeds, May* 30, 1809.

